# Argon and Argon–Oxygen Plasma Surface Modification of Gelatin Nanofibers for Tissue Engineering Applications

**DOI:** 10.3390/membranes11010031

**Published:** 2021-01-02

**Authors:** Abolfazl Mozaffari, Mazeyar Parvinzadeh Gashti, Mohammad Mirjalili, Masoud Parsania

**Affiliations:** 1 Department of Textile and Polymer Engineering, Yazd Branch, Islamic Azad University, Yazd 14515-775, Iran; mozaffari@iauyazd.ac.ir (A.M.); dr.mirjalili@iauyazd.ac.ir (M.M.); 2 Research and Development Laboratory, PRE Labs Inc., #100-2600 Enterprise Way, Kelowna, BC V1X 7Y5, Canada; 3 Department of Microbiology, Faculty of Medicine, Tehran Medical Sciences, Islamic Azad University, Tehran 19395/1495, Iran; masoud_parsania@yahoo.com

**Keywords:** electrospinning, gelatin, plasma activation, fibroblast cells, tissue engineering

## Abstract

In the present study, we developed a novel approach for functionalization of gelatin nanofibers using the plasma method for tissue engineering applications. For this purpose, tannic acid-crosslinked gelatin nanofibers were fabricated with electrospinning, followed by treatment with argon and argon–oxygen plasmas in a vacuum chamber. Samples were evaluated by using scanning electron microscopy (SEM), atomic force microscopy (AFM), attenuated total reflection Fourier transform infrared (ATR-FTIR) spectroscopy, contact angle (CA) and X-ray diffraction (XRD). The biological activity of plasma treated gelatin nanofibers were further investigated by using fibroblasts as cell models. SEM studies showed that the average diameter and the surface morphology of nanofibers did not change after plasma treatment. However, the mean surface roughness (RMS) of samples were increased due to plasma activation. ATR-FTIR spectroscopy demonstrated several new bands on plasma treated fibers related to the plasma ionization of nanofibers. The CA test results stated that the surface of nanofibers became completely hydrophilic after argon–oxygen plasma treatment. Finally, increasing the polarity of crosslinked gelatin after plasma treatment resulted in an increase of the number of fibroblast cells. Overall, results expressed that our developed method could open new insights into the application of the plasma process for functionalization of biomedical scaffolds. Moreover, the cooperative interplay between gelatin biomaterials and argon/argon–oxygen plasmas discovered a key composition showing promising biocompatibility towards biological cells. Therefore, we strongly recommend plasma surface modification of nanofiber scaffolds as a pretreatment process for tissue engineering applications.

## 1. Introduction

Since the mid-1990s, researchers have considered the potential production and implementation of nanofibers in various engineering applications. Electrospinning has been able to generate continuous fibers with diameters ranging from submicron down to the nanometer. As one-dimensional materials, nanofibers and nanowires have also been widely recognized in biomedical engineering due to the high surface area to volume ratio, nano-porosity, good absorption, biocompatibility, biodegradability, good breathability and mass transport properties [1,2,3,4]. In addition, flexibility of electrospinning process allows fabrication of continuous nanofibers for a various range of applications, including electronics and energy, textiles and protective clothing, sensors, energy storage and filtration [5,6,7].

Gelatin, as one of the main biopolymers, has been widely researched in tissue engineering through facile and effective electrospinning procedures. The ease of spinnability of this biomacromolecule resulted in morphologically uniform nano-fibrous scaffolds [5]. The aims of these studies have been accomplished in different tissue engineering applications, including cartilage, skin, vascular and hamstring systems [8,9,10]. In general, gelatin nanofibers can be achieved in relatively mild solvents, such as acetic acid/water and ethanol/formic acid/water solutions [5,10,11,12]. However, the main challenge in the application of gelatin nanofibers is their high solubility in water. In this regard, chemical and enzymatic cross-linking have been widely considered by researchers to improve their mechanical properties and preserve their morphology [13,14,15,16]. In addition, the toxicity of cross-linkers should be taken into consideration for biomedical applications. The majority of studies have demonstrated that cross-linked gelatin nanofibers can be produced via exposure to glutaraldehyde vapor, genipin, 1-ethyl-(3-3-dimethylaminopropyl) carbodiimide hydrochloride (EDC) and tannic acid [17,18,19]. It was reported that tannic acid crosslinked nanofibers were not only able to maintain the physical structure in water, but they can also act as antioxidants with antimicrobial functionality against different microorganisms including bacteria, viruses and fungi [20].

One of the most available approaches to modify the surface of polymers and fibers is the plasma activation method [21,22,23,24,25]. Many research works have focused on this process to increase the interaction of polymers with different materials. However, very few studies have been made to improve the bioactivity of nanofibers for tissue engineering applications [26,27,28]. Cheng et al. functionalized poly(L-lactide) micro-fibrous scaffolds with argon or argon-NH_3_/H_2_ plasmas to enhance affinity toward bovine aorta endothelial and bovine smooth muscle cells [29]. A similar study was conducted by Yoshida et al. to enhance the biocompatibility of polymer surfaces for controlled drug release purposes [30]. Indeed, very few studies on immobilization of gelatin on different plasma functionalized nanofibers are worthy of mention. Ma et al. activated polycaprolactone (PCL) nanofibers with air plasma grafted with gelatin. The resultant nanofibers had higher endothelial cell compatibility for blood vessel tissue engineering [31]. Studies have shown that fabrication of cationized gelatin on oxygen plasma treated poly(lactide-co-glycolide) can improve the affinity toward fibroblast cells [32]. Furthermore, this process introduced carboxylic acid groups on polylactic acid (PLLA) electrospun nanofibers that resulted in an improvement in immobilization of cationized gelatin. The final nanofibers were promising candidates for cartilage tissue generation [33]. Recently, Omrani and her group modified the surface of polyether ether ketone with gelatin in conjunction with oxygen plasma for bone related injuries [34]. Ghorbani et al. immobilized gelatin on the oxygen plasma-treated PCL that resulted in tunable pore structures for wound healing and skin tissue engineering [35]. However, to the best of our knowledge, there are no studies on plasma functionalization of gelatin nanofibers and evaluation of their bioactivities. Attempts were made in our research on argon and argon–oxygen plasma surface modification of gelatin nanofibers for tissue engineering applications.

## 2. Materials and Methods

Gelatin powder (type A, Bio Reagent with code G1890 from porcine skin), tannic acid and acetic acid (66%) were purchased from Sigma Aldrich.

According to the previous studies, we used acetic acid as a solvent for electrospinning of gelatin [29,36]. A solution containing 15% *w*/*v* gelatin and 5% *w*/*v* tannic acid was prepared in 10 mL acetic acid. Then, it was stirred for 4 h at 30 °C to achieve a homogenous solution. The electrospinning process was conducted at 15 kV voltage, and the distance of needle tip to metallic plate collector was set at 15 cm with a feeding rate of 0.6 mL/h. The electrospinning apparatus was from Fanavaran nano-meghyas Co. (Tehran, Iran). The collected nanofibers were then placed in a vacuum oven for 3 h at 45 °C to obtain a fully cross-linked scaffold.

Electrospun nanofiber scaffolds were treated in a plasma chamber (argon and argon–oxygen gas mixtures) for 90 s by using PF-200 plasma DBD device (Nik Fanavaran Plasma Co., Tehran, Iran). For pure argon gas, the flow rate was set at 2 L/min. On the other hand, we used an identical ratio for argon–oxygen gas mixtures at 2 L/min. The AC voltage was applied at 10 kV, and the distance between the sample and nozzle was 3 mm.

To evaluate the performance of the plasma modification process on the crosslinked gelatin for tissue engineering applications, gelatin films were prepared from the same electrospinning solution and were treated with argon and argon–oxygen plasmas. In this regard, primary human dermal fibroblast cells, derived from human skin fibroblasts (Royan Institute, Tehran, Iran), were cultured in Dulbecco’s Modified Eagle’s Medium (DMEM; Biosera, England) supplemented with 10% fetal bovine serum (FBS; Gibco, Belgium), 100 IU/mL penicillin and 100 μg/mL streptomycin. The cells were maintained at 37 °C in 5% CO_2_ condition. Then, cells (fourth passage) were cultivated on gelatin films with a size of 10 mm × 10 mm, by using a microscope slide cover glass (22 mm × 22 mm). For this purpose, we utilized 6-well cell culture plates with a cell seeding density of 10,000 cells/cm^2^ of medium. After culturing, cells were grown for 24 h and then used for microscopy imaging. We utilized an image processor (ImageJ) to count the average cell numbers per captured image. Three images from different areas were used and the average cell numbers were measured for each sample. The morphology of crosslinked gelatin nanofibers was investigated by SEM (LEO1455VP, Cambridge, England). For this purpose, samples were sputter-coated with an Au layer under vacuum conditions, prior to the microscopic assessment. We used a pumping system along with a coating thickness controller of MTM-20 with a sputtering power of 30 W. The coating thickness and the target-to-substrate distance were 10 and 50 mm, respectively. The SEM working distance was 9 mm for all samples and two magnifications of ×5 K and ×10 K (K = 1000) were used. The SEM operated at a 25 kV accelerating voltage. The average diameter of various nanofibers were measured by ImageJ. We used five measurements and the mean values were reported. AFM was utilized to determine the surface topography and the roughness of nanofibers on a contact mode of Scanning probe microscope (SPM) microscope (Park Scientific Instrument, Auto Probe CP Model, Korea). For this purpose, the root mean square (RMS), as the most important parameter for roughness characterization, was calculated according to Equation (1):

(1) $$RMS=\sqrt{\frac{\sum_{n-1}^{N}\left(Zn-Zm\right)2}{N-1}},$$

where Zn is the height measurement of pixel *n* (from a total of *N* = 256 × 256 pixels), and Zm is the mean height. The chemical properties of nanofibers were assessed by the FTIR spectroscopy (Thermo Nicolet NEXUS 870 FTIR from Nicolet Instrument Corp., Madison, WI, USA). The spectrophotometer was set up with a single reflection ATR accessory for reflection mode and measurements were conducted over a range of 500–4000 cm^−1^ at a resolution of 5 cm^−1^. Wettability of the scaffolds were examined using a water contact angle system supported by video camera equipment (Perkin Elmer Spectrum RX-1, Waltham, MA, USA). XRD of gelatin nanofibers was evaluated from wide-angle X-ray diffractograms recorded with a Philips X’Pert Pro Multipurpose X-ray Diffractometer operating at 40 mA. Ni-filtered Cu Ka radiation generated at 40 kV (k = 0.1542 nm) and the measured angle ranged from 4 to 70°, with the scan speed of 1°/min.

## 3. Results and Discussion

### 3.1. Microscopic Evaluation of Plasma Treated Nanofibers 

**Figure 1:**
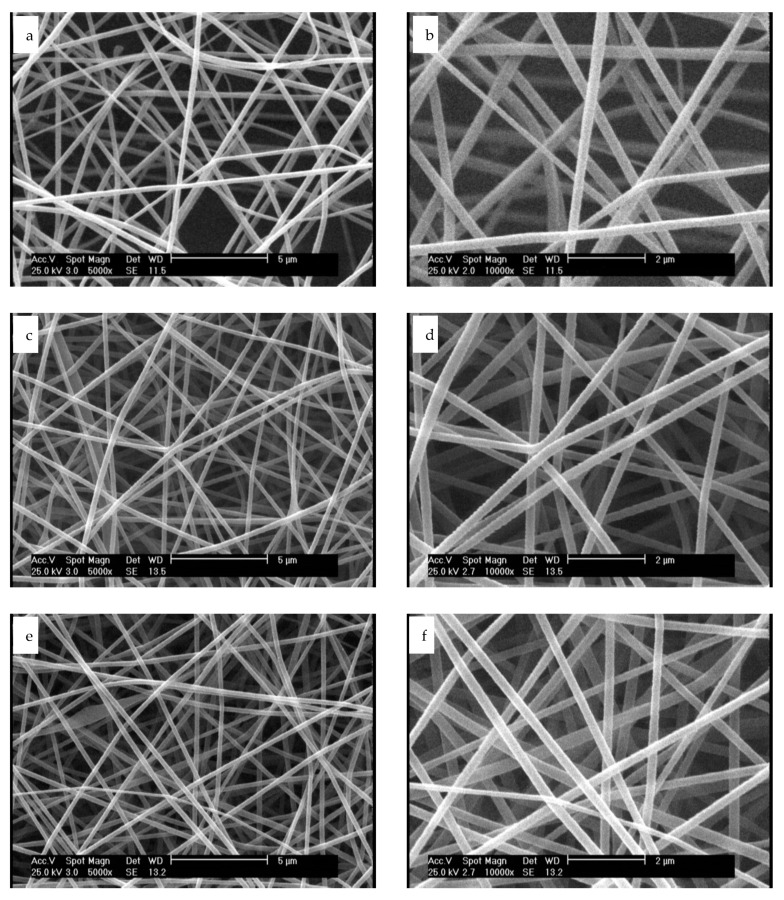
SEM images of: (**a**) nanofibers before plasma treatment at ×5000, (**b**) nanofibers before plasma treatment at ×10,000, (**c**) nanofibers after treatment with argon plasma at ×5000, (**d**) nanofibers after treatment with argon plasma at ×10,000, (**e**) nanofibers after treatment with argon–oxygen plasma at ×5000 and (**f**) nanofibers after treatment with argon–oxygen plasma at ×10,000.

**Figure 2:**
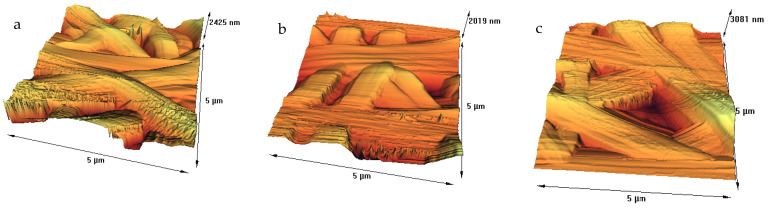
Atomic force microscopy (AFM) images of: (**a**) nanofibers before plasma treatment, (**b**) nanofibers after treatment with argon plasma and (**c**) nanofibers after treatment with argon–oxygen plasma.

We used acetic acid for dissolving gelatin in water, owing to the fact that it prevents the bead-like non-uniform electrospun gelatin nanofibers [37]. SEM and AFM of tannic acid crosslinked nanofiber scaffolds before and after plasma process were assessed, and results are displayed in Figure 1 and Figure 2. 

According to Figure 1a,b, untreated nanofibers are uniform before plasma treatment, with no beads and an average diameter of 300 ± 28 nm. Our previous study on fabrication of tannic acid crosslinked gelatin nanofibers revealed that a balance between the electrostatic repulsion, surface tension and viscoelastic properties were required to generate uniform nanofibers. The higher tannic acid content in gelatin bath, the higher the average diameter of nanofiber will be [36]. After plasma treatment with argon and argon–oxygen gases, the average diameter of nanofibers were not considerably changed (294 ± 37 nm and 291 ± 45 nm, respectively). Moreover, no considerable change was observed on the nanofiber surfaces. Shen et al. evaluated the role of oxygen plasma treatment on adhesion of cationized gelatin onto poly(lactide-co-glycolide) films. According to their SEM results, plasma was not only able to promote a uniform distribution of cationized gelatin on the biopolymer films, but it could also generate a mesh-like surface [32]. On the other hand, argon or argon–ammonia–hydrogen plasmas did not affect the surface morphology of PLLA microfibers [29]. Our SEM results are consistent with this study. The effect of solvent systems on the morphology of gelatin nanofibers has been extensively studied elsewhere [38,39]. Choktaweesap et al. demonstrated that electrospinning of 15–29% *w*/*v* gelatin solutions in acetic acid generated beads, beaded fibers and smooth fibers, depending on the concentration changes. On the other hand, dimethyl sulfoxide and ethylene glycol can reduce the diameter of fibers with smooth surface effects [38]. Horuz and Belibağlı employed Taguchi’s methodology to optimize electrospinning of gelatin nanofiber with acetic acid. The optimum process conditions were fixed in 20% acetic acid under a voltage of 18 kV, a flow rate of 15 μL min^−1^ and a distance of 12.5 cm [39].

We further investigated the surface properties of nanofibers with AFM due to the fact that it generally provides accurate measurements on surface topography. We also extracted the results from the AFM test, and roughness parameters are shown in Table 1. The surface of the untreated fibers was smooth, with the RMS being about 5.1 nm, as calculated from Figure 2a. The RMS for argon and argon–oxygen plasma treated samples (Figure 2b,c) were 31.54 and 27.76 nm, respectively. An increase in the surface roughness of nanofibers after plasma treatment can be attributed to the bombardment of energetic particles, including electrons, ions, radicals, neutrals and excited atoms/molecules [21,22,23]. Argon–oxygen plasma can result in chemical etching by bond breakage, chain scission, chemical degradation and surface oxidation, while argon plasma is an inert process and can physically etch nanofibers by removal of low molecular weight fragments [23,24,25]. Our previous studies demonstrated similar results on various films and fibers. However, a research group did not observe roughening effect on the plasma treated electrospun PLLA fibers due to stretched polymer chains oriented parallel to the longitudinal fiber direction [29].

### 3.2. Chemical Characteristics of Plasma Treated Nanofibers

**Figure 3:**
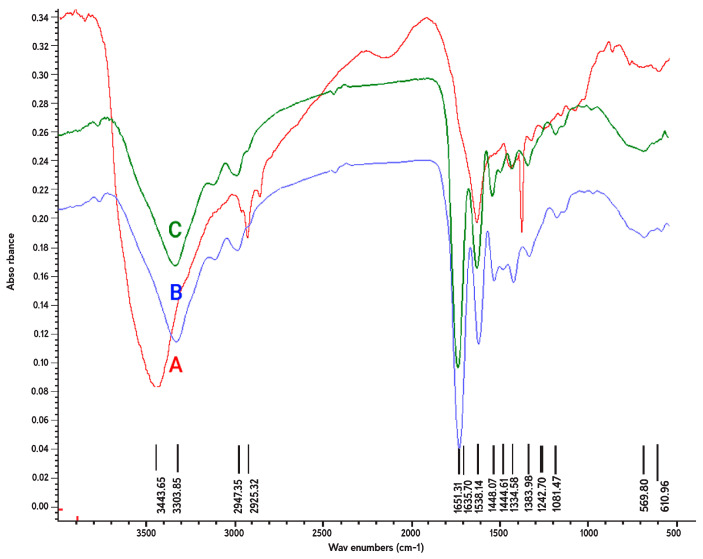
The attenuated total reflection Fourier transform infrared (ATR-FTIR) spectra of (**A**) crosslinked gelatin nanofibers before plasma treatment, (**B**) argon plasma treated nanofibers and (**C**) argon–oxygen plasma treated nanofibers.

Figure 3 illustrated the ATR-FTIR spectra for the crosslinked gelatin nanofibers, before and after treatment with argon and argon–oxygen plasmas. We also represented the major peaks for fabricated samples in Table 2. The FT-IR spectrum of gelatin (Figure 3A) indicated several bands for N–H stretching of amide bond at 3443 cm^−1^, C–H stretching at 2925 cm^−1^ and aromatic C–H bending at 610 cm^−1^ [38]. The amide II peak at 1538 cm^−1^ was attributed to N–O stretching of gelatin macromolecules [39,40,41]. The amide III band was attributed to the combination of N–H in plane bending, C–N stretching vibrations and N–H out-of-plane wagging at 1334 cm^−1^. The C–O stretching bands also appeared at 1242 and 1300 cm^−1^ [42,43,44,45]. 

After plasma treatment of nanofibers, several changes were observed in the ATR-FTIR spectrum of gelatin, including shifting of two peaks from 3443 (amide A) and 2925 to 3303 and 2947 cm^−1^, respectively; appearance of three peaks at 1651 (amide I), 1448 and 1441 cm^−1^ (amide II); and disappearance of a peak at 1300 cm^−1^ (amide III). Appearance of new peaks in the spectrum can be possibly due to generation of COO or carbonyl groups in nanofibers due to plasma ionization. In addition, plasma process could have a major effect on several peaks in the amide A, amide II and amide III regions in gelatin. Shen et al. used FTIR spectroscopy to detect gelatin on oxygen plasma treated poly(lactide-co-glycolide) films. They found that gelatin can be effectively anchored onto poly(lactide-co-glycolide) due to oxidation [32]. Similar to our study, Ghorbani et al. recently investigated the effect of oxygen plasma treatment on PCL nanofibers. They observed oxygen-containing functional groups, including carbonyl groups and –COO bonds at 1272 cm^−1^ and 1420 cm^−1^, respectively [35]. We should mention that there was not considerable difference between the ATR-FTIR spectra of argon and argon–oxygen plasma treated nanofibers.

### 3.3. Water Contact Angle Properties of Nanofibers 

The importance of hydrophilic properties of gelatin scaffolds in tissue engineering applications has been reported in several studies [28,37]. We measured the water contact angle (CA) of nanofiber scaffolds by using the droplet size 0.5 mL. Three samples were selected in each experiment and the mean values of CA were reported. In general, gelatin has hydrophilic properties, and it displays excellent wettability in comparison with other polymers [46,47,48,49]. As can be seen in Table 3, the gelatin nanofiber scaffold had a mean CA of 20.65°. After argon plasma treatment, the mean CA value decreased to 14.6°, which can be due to the changes on the surface properties of nanofibers. However, we interestingly observed that argon–oxygen plasma treatment resulted in a highly hydrophilic gelatin nanofiber and the water droplet was completely absorbed into the scaffold. The introduction of polar groups onto the nanofiber surface by argon–oxygen plasma could lead to a completely hydrophilic scaffold, which was confirmed in our ATR-FTIR spectroscopy assessment. 

Our results are also in agreement with several studies on plasma functionalization of polymers for tissue engineering purposes [29,30,31,32,33,34]. Ma et al. found that air plasma increases the hydrophilicity of PCL and improves the interactions with gelatin coating [31]. In two studies, oxygen, argon and argon–ammonia–hydrogen plasmas were able to decrease the CA of PLLA nanofibers from values higher that 110° to 15°, 85° and 0°, respectively. These groups confirmed generation of carboxylic acid groups on PLLA after plasma treatments [29,33]. Similar hydrophilic action was also observed for diamond like carbon films [30] and polyether ether ketone samples [34]. In our recent research, we compared the effect of crosslinking of gelatin with tannic acid on CA properties. Tannic acid reduced the CA due to crosslinking of functional groups at the scaffold surface and the presence of aromatic rings in tannic acid [36]. Our group previously investigated the moisture absorption of polyester fibers after air plasma treatment. Results postulated that air plasma increased the moisture absorption from 4.4% to 6.2% due to generation of polar groups on the fiber surfaces [21,22,23,24,25].

### 3.4. XRD Analysis of Nanofibers 

**Figure 4:**
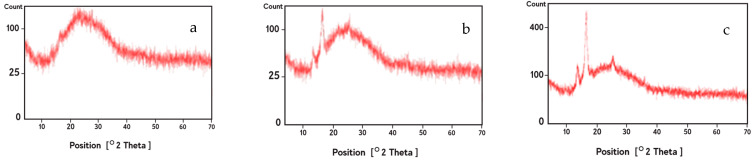
XRD analysis of (**a**) crosslinked gelatin nanofibers scaffolds before plasma treatment, (**b**) nanofibers after argon plasma treatment and (**c**) nanofibers after argon–oxygen plasma treatment.

The XRD patterns for the crosslinked gelatin nanofibers before and after treatment with argon and argon–oxygen plasmas are depicted in Figure 4. Gelatin is a partially crystalline biopolymer with a relatively intensive peak at 2θ = 8° (d_101_ = 11.08 Å) and a broad peak at 2θ = 24° (d_101_ = 4.01 Å). These characteristic peaks are related to the triple-helical crystalline structure of gelatin biomacromolecules. However, we did not observe the intensive peak at 2θ = 8° due to addition of tannic acid as crosslinker in the fiber processing (Figure 4a). This observation was previously stated by Peña et al. [50].

After argon (Figure 4b) and argon–oxygen plasma (Figure 4c) treatment of nanofibers, two peaks appeared at 2θ = 15° and 17°, demonstrating a new biomacromolecular orientation in plasma treated nanofibers. These peaks were more intensified in the argon–oxygen plasma sample due to the key role of oxygen gas in the crystallinity of gelatin scaffolds. An increase in the peak intensities for argon–oxygen plasma treated nanofibers can be attributed to the presence of bigger crystallites or an increase in the crystallinity fraction. In agreement to our results, two research groups recently reported appearance of new peaks in the XRD spectra of polyamide fibers after plasma activation [51,52]. 

### 3.5. Morphological Characterization of Cells on Nanofiber Scaffolds

**Figure 5:**
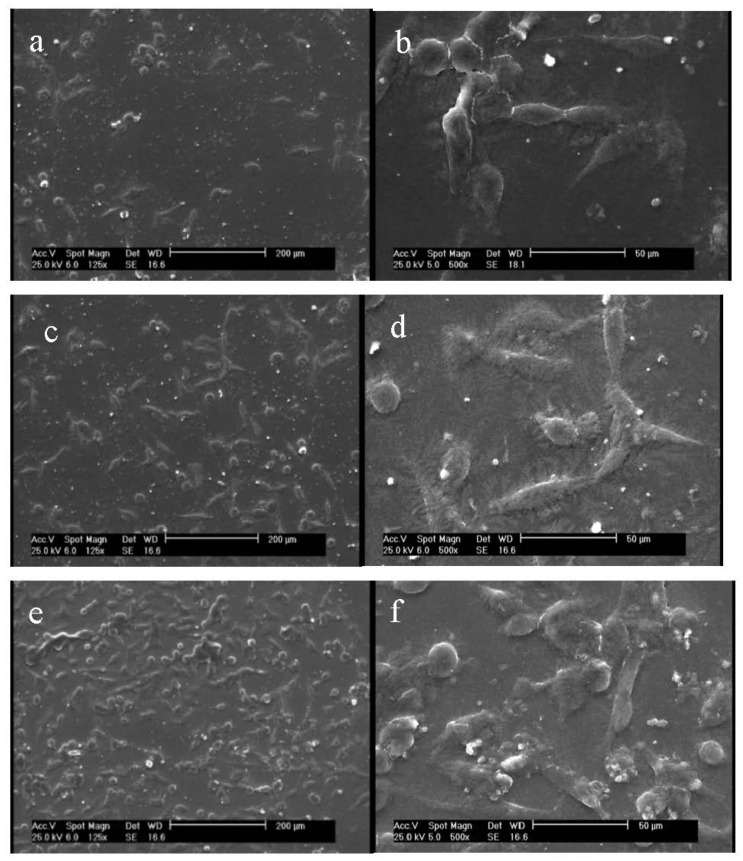
SEM images of cultured fibroblast cells on the gelatin films: (**a**) cells on the untreated gelatin film at ×125, (**b**) cells on the untreated gelatin film at ×500, (**c**) cells on the argon plasma treated gelatin film at ×125, (**d**) cells on the argon plasma treated gelatin film at ×500, (**e**) cells on the argon–oxygen plasma treated gelatin film at ×125 and (**f**) cells on the argon–oxygen plasma treated gelatin film at ×500.

Prepared gelatin films, before and after argon and argon–oxygen plasma treatments, were assessed regarding their ability to interact with fibroblast cells. The morphology of cells were observed by using SEM, and the images are illustrated in Figure 5. As can be seen from Figure 5b, fibroblast cells had their natural spindle shape on the gelatin film before plasma process (average cell number: 85 ± 6). After argon plasma treatment, the number of fibroblast cells increased on gelatin (average cell number: 96 ± 8; Figure 5b). We interestingly observed further increase in the number of these cells on the argon–oxygen plasma treated gelatin film (average cell number: 145 ± 9; Figure 5c). This is attributable to the fact that more polar groups were generated on the argon–oxygen plasma treated gelatin, and the affinity toward fibroblast cells was increased. This result is in agreement with water CA properties. Notably, there was no considerable difference in the shape of cells on argon and argon–oxygen plasma treated films. Shen et al. noted that deposition of gelatin layer on the oxygen plasma treated poly(L-lactide-co-glycolide) film could accelerate adhesion of fibroblast cells. The reason was generation of hydrophilic functional groups at the film surface [32]. Cheng et al. stated that the enhancement of cell adhesion and spreading on plasma treated surfaces was mainly due to improvements in hydrophilicity and the incorporation of O and N functional domains. Indeed, they did not find any relation between the cell numbers and the surface roughness after plasma treatment. Moreover, they depicted an increase in the cell spreading on argon–ammonia–hydrogen plasma treated surfaces, in comparison with the argon plasma-treated ones. Similar results can be seen in our study on argon and argon–oxygen plasma treated gelatin films [29]. We should also highlight research by Chandrasekaran et al. that is in agreement with our research. They demonstrated that poly(L-lactic acid)-co-poly(ε-caprolactone)/gelatin scaffolds exhibited significant increase in fibroblasts proliferation, morphology and secretion of collagen after plasma treatment [53].

## 4. Conclusions

We fabricated crosslinked gelatin nanofibers for treatment with argon and argon–oxygen plasma procedures. The main aims of this study were to evaluate the surface properties and bio-functionality for tissue engineering applications. We observed that the RMS of nanofibers were increased after plasma treatment. However, the argon plasma treated sample showed a rougher surface in comparison with the argon–oxygen plasma treated one. Surface roughness of samples was related to the changes in the chemical properties of nanofibers. Our results were further approved by CA evaluation test. The CA of argon plasma treated samples decreased in comparison with the untreated nanofibers. However, due to surface oxidation of argon–oxygen plasma treated nanofibers, the CA value was 0° and the sample was completely hydrophilic. We noted the appearance of a new peak in the XRD spectra of plasma treated nanofibers due to changes in the crystallinity. Finally, the number of fibroblast cells increased on the argon and argon–oxygen plasma treated gelatin films, and these cells maintained their normal shape.

Overall, the cooperative interplay between plasma surface modification and gelatin nanofiber fabrication resulted an improvement in the surface functionality, biocompatibility and bioactivity toward fibroblast cells. Here, we conclude that argon and argon–oxygen plasma methods are likely to play an important role in tissue engineering applications. Further studies are necessary in order to investigate the suitability of plasma for activation of other polymers in biomedical engineering and tissue regeneration.

## Figures and Tables

**Table 1:** Roughness values as a function of plasma treatment on crosslinked gelatin nanofibers.

Samples	Plasma Treatment Time (s)	Maximum Peak Height, R_a_ (nm)	Maximum Valley Depth, R_z_ (nm)	Average Peak-to-Valley Height R_q_ (nm)	RMS (nm)
Untreated nanofibers	0	6.585	276.8	47.48	5.1
Argon plasma treated nanofibers	90	1.635	22.95	11.79	31.54
Argon–oxygen plasma treated nanofibers	90	1.056	20.73	10.63	27.76

**Table 2:** The main ATR-FTIR peaks observed in crosslinked gelatin nanofibers before and after plasma treatment.

Crosslinked Gelatin Nanofibers before Plasma Treatment		Crosslinked Gelatin Nanofibers after Argon Plasma Treatment		Crosslinked Gelatin Nanofibers after Argon/Oxygen Plasma Treatment	
Peak Position (cm^−1^)	Band Assignment	Peak Position (cm^−1^)	Band Assignment	Peak Position (cm^−1^)	Band Assignment
610–669	–CH bending	610–669	–CH bending	610–669	–CH bending
1242	C–O stretching/Amide III	1242	Amide III	1242	Amide III
1300	C–O stretching	-	-	-	-
1334	C–N stretching/Amide III	1334	Amide III	1334	Amide III
-	-	1444	Amide II	1444	Amide II
-	-	1448	Amide II	1448	Amide II
1538	N–O stretching/Amide II	1538	Amide II	1538	Amide II
-	-	1651	C=O stretching	1651	C=O stretching
2925	–CH stretching	-	-	-	-
-	-	2947	–CH stretching	2947	–CH stretching
-	-	3303	Amide A	3303	Amide A
3443	O–H Stretching/Amide A	-	-	-	-

**Table 3:** Water contact angle of (**a**) crosslinked gelatin nanofibers scaffolds before plasma treatment, (**b**) nanofibers after argon plasma treatment and (**c**) nanofibers after argon–oxygen plasma treatment.

Samples	Mean Contact Angle, CA (°)	Camera Image
Untreated nanofibers	20.65	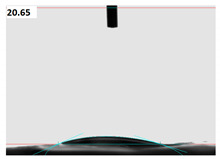
Argon plasma treated nanofibers	14.6	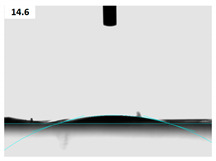
Argon–oxygen plasma treated nanofibers	0	Completely hydrophilic
